# Pelvic Asymmetry and Stiffness of the Muscles Stabilizing the Lumbo–Pelvic–Hip Complex (LPHC) in Tensiomyography Examination

**DOI:** 10.3390/jcm14072229

**Published:** 2025-03-25

**Authors:** Karol Bibrowicz, Katarzyna Ogrodzka-Ciechanowicz, Zuzana Hudakova, Tomasz Szurmik, Bartosz Bibrowicz, Piotr Kurzeja

**Affiliations:** 1 Science and Research Center of Body Posture, Kazimiera Milanowska College of Education and Therapy, 61-473 Poznan, Poland; bibrowicz@wp.pl; 2 Institute of Clinical Rehabilitation, Faculty of Motor Rehabilitation, University of Physical Culture in Krakow, 31-571 Krakow, Poland; 3 Faculty of Health, Catholic University, 034 01 Ružomberok, Slovakia; zuzana.hudakova@ku.sk; 4 Department of Health Care Studies, College of Polytechnics, 586 01 Jihlava, Czech Republic; 5 SNP Central Military Hospital, Faculty Hospital, 034 01 Ružomberok, Slovakia; 6 Faculty of Arts and Educational Science, University of Silesia, 43-400 Cieszyn, Poland; info@orto-med.com.pl; 7 Research and Development Center Legia Lab, Legia Warszawa, 00-449 Warszawa, Poland; bartosz.bibrowicz@gmail.com; 8 Institute of Health Sciences, Academy of Applied Sciences, 34-400 Nowy Targ, Poland; piotrkurzeja@op.pl

**Keywords:** tensiomyography, inclinometry, asymmetry, posture, muscle stiffness

## Abstract

**Background:** The pelvic girdle is an important component of the human stabilization system, both during the maintenance of an upright standing position and during motor activities. Frequent functional and structural asymmetries within it can affect the structure and function of many organs and systems of the human body. The mechanism of their occurrence is not fully explained. The objective of the present study was to verify the hypothesis regarding the relationship between the value of pelvic asymmetry and the functional state of muscles that stabilize the lumbo–pelvic–hip complex, as measured by changes in their stiffness. **Methods**: The study group consisted of 40 young women aged from 19 to 29 years. The observational cross-sectional study incorporated the following elements: an interview, an anthropometric test, an inclinometric assessment of the magnitude of hip girdle rotation utilizing a duometer and tensiomyography. **Results**: Analysis of the variables examined in subjects with symmetric or rotated pelvises did not show significant differences between the studied sides in the two groups. Evaluation of associations between the magnitude of pelvic rotation and tensiomyography findings showed that with increased pelvic rotation, the stiffness of the back extensor muscles and the rectus thigh muscles increased only slightly bilaterally, and the contraction rate of the rectus abdominis and biceps thigh muscles decreased. **Conclusions**: The results of the tensiomyography study did not unequivocally demonstrate that changes in pelvic symmetry in the transverse plane are associated with dysfunction of the muscles that stabilize the pelvic girdle.

## 1. Introduction

Symmetry is quite rare in the human body. However, asymmetries are commonly observed. They can be both morphological and functional. They are connected with ontogenetic development, lateralization, the character of work, sport activities or lasting diseases [1,2,3,4,5,6,7,8,9,10]. Mechanisms are described in which diseases can be primary causes of pelvic asymmetry or the opposite—where they can lead to illnesses and ailments [11,12,13,14,15,16]. Sometimes, body asymmetries among children and adolescents can have a direct impact on posture development and the occurrence of spinal deformations as well as on postural stability [17]. The role of physicians, anthropologists, biomechanics and physiotherapists is to evaluate these asymmetries and determine their origin, their influence on the patient’s health, and—if they can cause overloads and, consequently, degenerative changes and pain—to develop preventive strategies and therapeutic methods. Asymmetries in the pelvic girdle area seem to be worth particular attention. They are connected with, among others, the pelvic girdle, which functions as a buffer in transferring biomechanical loads, in locomotion or in back pains [11,13,15,18]. So far, the mechanisms of asymmetries have not been fully explained [19,20]. Those who search for the causes and, consequently, methods of therapy for pelvic asymmetries often assume that musculoskeletal asymmetries often coexist with myofascial disfunctions [13,16,18]. Since changes occur in the orientation of the selected skeletal landmarks and with them, changes in the location of muscle attachments and the fascial system, then some changes in muscle functions, especially tension, should occur as well [14,16]. Many popular therapeutic methods based on the correction of the myofascial system, like PIR, myofascial release or Rolfing [19,21,22], involve different types of activities to restore myofascial balance and, thus, eliminate the overload factors.

Different forms of asymmetry, regarding both morphology and functioning, are much more common. This is true also for the pelvic girdle area. Pelvic asymmetry is observed in the orientation of pelvic bones in the frontal, sagittal and transverse planes [11,20,23]. Asymmetries can be of the primary type, with a functional background, as well as the secondary type, resulting from different diseases like scoliosis or leg length discrepancy [12,13,18]. One of the symptoms of pelvic asymmetry is its rotation. Lewit [19] describes it as a contralateral asymmetry of both pelvic bones, which involves elevation of the posterior superior iliac spine with simultaneous lowering of the anterior superior iliac spine on the same side. Most often, the anterior superior iliac spines are lowered on the right side. This mechanism leads to an oblique positioning of the sacral bone, which results in posture asymmetry and asymmetrical transfer of dynamic loads from the lower limbs to the trunk. This may cause a reflex increase in the muscle tone which, in turn, leads to an apparent shortening of one leg. So far, the pelvic rotation mechanism has not been fully explained. Numerous studies revealed asymmetric tissue adaptations within the bone and muscle systems in persons engaged in sports, which are mainly unilateral [1,5]. This finding suggests the hypothesis that directional asymmetry can be interpreted as an asymmetric anatomical adaptation that occurs in response to unilateral, repetitive biomechanical loads [7,24]. According to the classic overload principle, anatomic adaptation of biological tissues occurs when these tissues are subjected to a level of stress that exceeds their typical stress threshold during routine daily activities [25]. The hypothesis posits that asymmetries of the pelvis can result in an inaccurate mechanical distribution of load on the body, thereby increasing the stress on the soft tissues in the lumbar region. These asymmetries have been linked to the onset of non-specific chronic back pain [15].

A question arises about effective prevention of pelvic asymmetries, and about the relationship between the asymmetry magnitude and stiffness of the muscles that stabilize the pelvic girdle. Assessment capabilities regarding the functional condition of tissues have improved greatly in the recent years. This is due to the development of research methods and tools which eliminate the shortcomings of palpation.

In particular, this may be the case when assessing muscle stiffness. Some studies have suggested a possible link between muscle stiffness and factors such as joint flexibility, postural balance and the risk of injury [26,27,28]. Furthermore, it has been suggested that an appropriate level of lower limb stiffness may be required to achieve optimal performance in activities such as running, jumping and hopping [29]. The measurement of muscle or tendon stiffness is believed to have several valuable applications. These applications include the detection of pathological changes, the monitoring of the effectiveness of rehabilitation programs, the direct assessment of athletic performance and the comparison of the effectiveness of different interventions for research purposes [30]. This refers to both evaluation of the surface muscles using a Myoton and tensiomyography (TMG), and examinations of more deeply located muscles by means of elastography [31,32,33,34,35,36,37,38]. TMG is a highly reliable method [32,33]. The examination is performed using a TMG device consisting of an electric stimulator, electrodes, a sensor which measures the size of the muscle belly displacement and the dedicated software [32].

The purpose of this study was an attempt to verify the hypothesis about the relationship between the value of pelvic asymmetry and the functional state of muscles stabilizing the lumbo–pelvic–hip complex, expressed as a change in their stiffness.

## 2. Materials and Methods

### 2.1. Study Design

This observational cross-sectional study adheres to the guidelines of the Helsinki Declaration and was conducted in compliance with the Strengthening the Reporting of Observational Studies in Epidemiology (STROBE) Statement: guidelines for reporting observational studies [39].

The observation was conducted as part of a long-term research program that was approved by the Bioethics Committee of the College of Physiotherapy in Wrocław (No. 1/2010, 25 June 2010).

### 2.2. Setting

This study was carried out between March and November 2020 at the Kazimiera Milanowska College of Education and Therapy in Poznań, Poland.

### 2.3. Participants

A total of 136 female students pursuing a degree in physiotherapy at the College of Education and Therapy in Poznań, Poland, were included in the study. The participants ranged in age from 19 to 29 years (X = 21.43, SD = 3.18). The survey revealed that none of the women were engaged in competitive sports.

The inclusion criteria were as follows:

- age between 19 and 29 years;
- right lateralization of the hand and foot;
- no discernible abnormalities were observed in the subject’s body composition;
- Body mass index (BMI) between 18.5 and 24.9.

The exclusion criteria were as follows:

- pain in the lumbo–pelvic–hip complex area or use of analgesics during the tests;
- pregnancy or menstrual phase;
- lack of written consent to take part in the study.

From the group that met the specified criteria, 40 women were selected at random to participate in the study. These women were divided into two groups of 20 individuals each, based on their pelvic symmetry as determined by a thorough evaluation. Twenty women with a symmetrical pelvis and 20 with symptoms of pelvic asymmetry were selected. The authors’ clinical typology of the pelvic region was used to assess pelvic symmetry [10,40].

### 2.4. Outcome Measures

The measurement of mass and height was conducted using a verified medical column scale, specifically the UNIWAG Professional Electronic Scales (UNIWAG, Krakow, Poland), which ranges from 100 to 200 cm for height measurement. The assessment of obesity level was determined using the BMI (body mass index) calculation.

**Figure 1:**
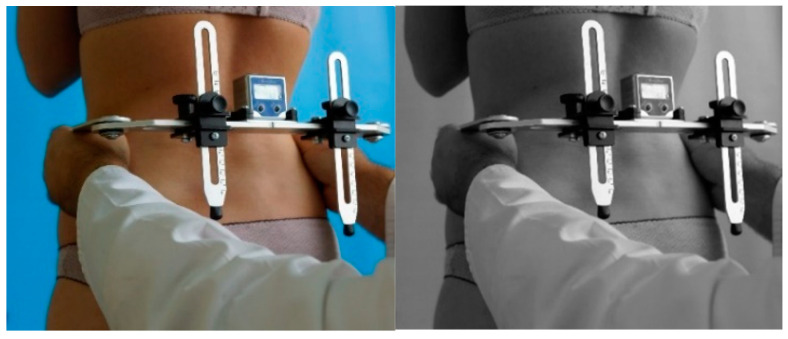
Measurement of the iliac crest angle using Duometr [own source].

The assessment of pelvic symmetry was based on the measurement of selected anthropometric points of the pelvis and lower limbs. The position of these landmarks in the pelvic girdle was determined using a scalable anthropometric leveler with an electronic inclinometer (DUOMETR, OPIW, Chrząstowice, Poland). This device enables measurements with 0.1° accuracy, and the authors assumed an accuracy of 1° (Figure 1).

The following indicators were subjected to analysis:

- ICA (iliac crest angle): the angle between the horizontal axis and the line formed by the apices of the iliac crests;
- ASISA (anterior superior iliac spine angle): the angle between the horizontal axis and the line formed by the anterior superior iliac spines;
- TMA (trochanter major angle): the angle between the horizontal axis and the line formed by the tops of the greater trochanters.

### 2.5. Intervention

All examinations were conducted in the morning to ensure consistent measurement conditions. Each participant was tested on three occasions, and the mean values were recorded. All inclinometric and TMG measurements were performed by the same experienced investigator.

**Figure 2:**
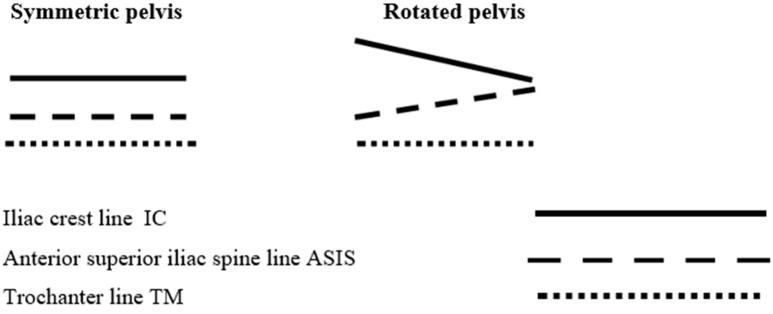
Graphic representation of pelvic asymmetry types [own source].

Pelvis asymmetry measure (position of selected LPHC landmarks):

During each examination, the rule was followed that the examined landmark was touched with the tip of the middle finger, and the measuring device arms were positioned strictly on the radial side of the middle finger. The materials collected were divided into groups according to the spatial location of the selected landmarks on the pelvic girdle, using the authors’ original clinical typology (Figure 2). The elements of the pelvic symmetry classification were chosen based on the analysis of the mutual position of lines running through the selected landmarks of the lumbo–pelvic–hip complex: 1. the line connecting the apices of the iliac crests (ICs); 2. the line connecting the anterior superior iliac spines (ASISs) and 3. the line connecting the apices of the greater trochanters of the femur (TMs) [10].

In view of the assumptions presented herein, respondents with a symmetric pelvis and asymmetric pelvis were identified in the analysis, while individuals with an oblique pelvis were not considered.

I. Symmetric pelvis (SP)

It was established that all lines ran in parallel to the floor, or the value of the analyzed angles, the ICA, ASISA and TMA, were <1°.

II. Asymmetric (rotated pelvis) (RP)

The iliac crest line and the anterior superior iliac spine line exhibit asymmetrical characteristics that vary with different factors; i.e., ICA and ASISA ≥ 1°. The trochanter major line runs parallel to the floor (TMA < 1°), or it runs in the same way as the anterior superior iliac spine line (TMA ≥ 1°) (Figure 2).

The rotation of the pelvis was determined based on the angle between the line of the apices of the iliac crests and the line of the anterior superior iliac spines.

**Figure 3:**
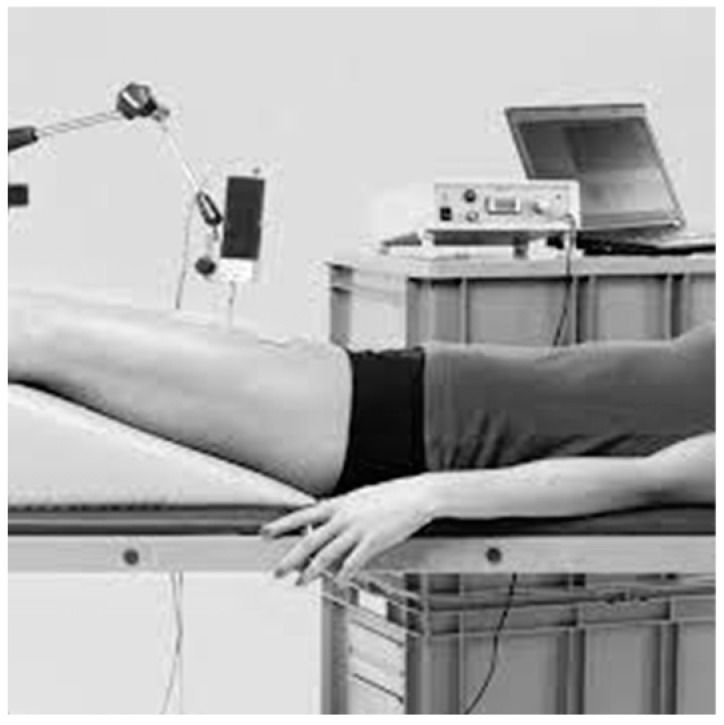
TMG of the rectus femoris [own source].

The Tensiomyography (TMG) test was executed using a TMG-BMC Ltd. (Ljubljana, Slovenia) system, designated as the TMG-BMC Science for Body Evolution system. The methodology of measuring individual muscles followed the procedures outlined by the device manufacturer. Electrodes were positioned in accordance with the SENIAM guidelines [41]. The following muscles were measured: the gluteus maximus, biceps femoris, rectus femoris, rectus abdominis, obliques abdominis ext. and the erector spinae. All measurements were made in a laboratory at a temperature of 22 ± 1 °C. During the measurements of the erector spinae, gluteus max. and biceps femoris, respondents remained in the prone position with ankle joints placed on a triangle foam pad. The muscles rectus abd., obliques abd. ext. and rectus fem. were examined in the supine position with legs bent and knees under the 20o angle. The tensiomyography test involves evaluation of the muscle response (time of response and displacement) to an electric stimulus. Two standard, self-adhesive, 2 mm thick ECOSTIM electrodes (EL.P.NWCS50.50.sq. Shiaoxing, China) were placed symmetrically, distally and proximally to the top of the TMG-S1 sensor (2.5 cm in each direction). A stimulator was used (EMF-Furlan I Co. doo, Ljubljana, Slovenia). The electric stimulation was provided with a single-twitch impulse of 1 ms in duration and 20 mA intensity, which was increased by 10 mA every 10–15 s until no further change in Dm was observed or the maximum power of the stimulator (110 mA) was reached. The cathode was placed proximally and the anode distally. The baseline parameters were obtained after two subsequent measuring protocols in a 5 min interval, and the mean values were used for the analysis (Figure 3).

**Figure 4:**
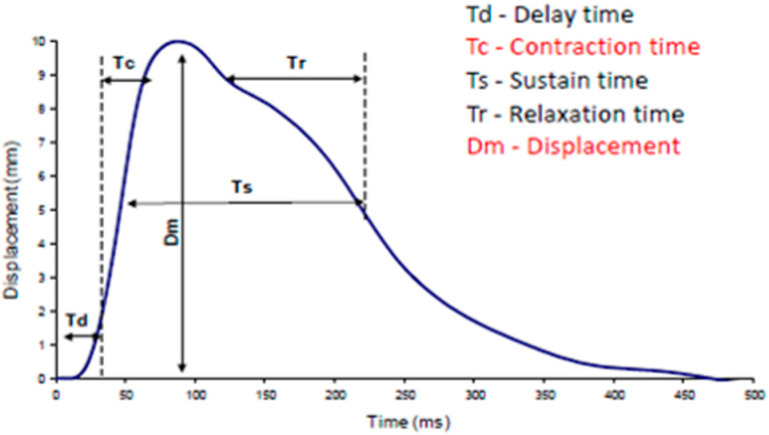
Example diagram of the muscle response to the electric stimulus [own source].

The values of displacement (Dm) describing muscle stiffness, as presented in the study, were obtained using the tensiomyographic method. It should be noted that these values represent the mean of measurements performed on the left and right side of the body (Figure 4).

### 2.6. Statistical Analysis

The statistical analysis of the material was conducted using the MedCalc package (MedCalc^®^ Statistical Software version 23.1.3 (MedCalc Software Ltd., Ostend, Belgium; https://www.medcalc.org; accessed on 3 Januarry 2025). The distributions of the variables were determined by means of the Shapiro–Wilk test. A standard descriptive analysis was presented using the mean values (X) and their standard deviations (SDs). The differences were calculated by means of Student’s t-test for independent groups. The relationships between the amount of pelvic rotation and the studied tensiomyography variables were assessed using Pearson’s correlation.

## 3. Results

**Figure 5:**
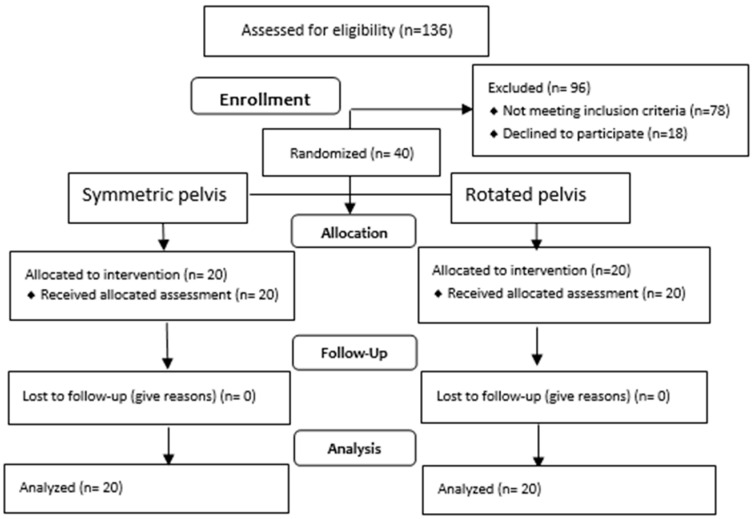
Flow diagram.

A total of 136 individuals were qualified for the tests, out of which only 40 met the eligibility criteria. The qualification process is presented in Figure 5. Table 1 presents the respondents’ anthropometric data.

**Figure 6:**
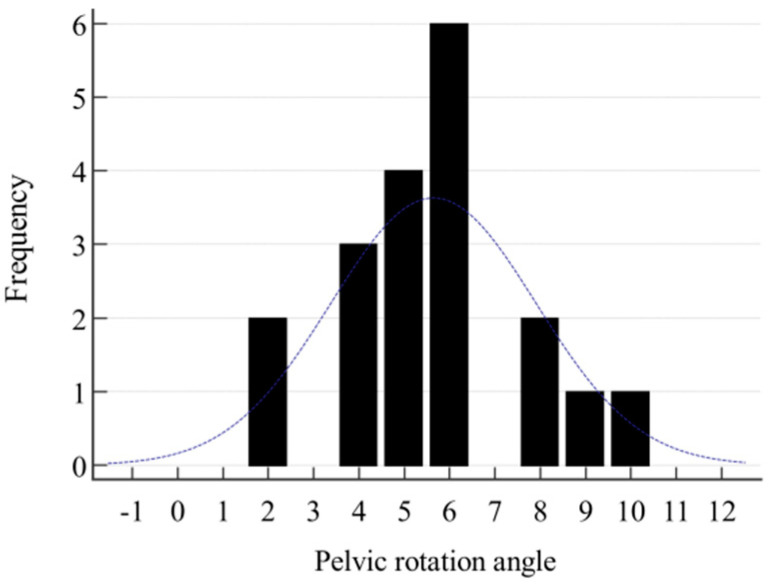
Distribution of the group according to the size of the pelvic rotation angle.

The results of the analysis showed a normal distribution of the studied variable in the respondents with a rotated pelvis. The mean magnitude of rotation, viewed as the angle between the line of the anterior superior iliac spines and the line of the apices of the iliac crests, was X = 5.6°, SD 2.09, with the results ranging 2–10°. The distribution of the group investigated is shown in Figure 6.

The descriptive analysis of displacement (Dm) in the whole group demonstrated a normal distribution for all the variables in question, similar for the right and left side. The analysis of differences, conducted using Student’s *t*-test for independent groups, did not show any statistically significant differentiation on any side (Table 2). A repeated analysis of variables, conducted in the groups identified based on the pelvis type, did not reveal any significant differences between the sides as well as between the results obtained for the symmetric pelvis and the rotated pelvis (Table 3).

Evaluation of correlations between the rotation magnitude and displacement (Dm) did not show any clear linear correlations between the variables studied. However, some correlations were observed. The Dm values indicated that with a growing rotation angle, the stiffness of the erector spinae and rectus femoris increases. This is true for muscles on both the right and left side (Table 4).

## 4. Discussion

The objective of the present study was to ascertain the existence of a reciprocal relationship between the magnitude of pelvic asymmetry, as indicated by the size of the angle between the straight lines passing through the anterior superior iliac spines and the tops of the iliac crest, and the stiffness of the stabilizing muscles of the LPH complex, as demonstrated by the magnitude of the radial displacement of the muscles under study following an electrical stimulus on tensiomyography (Dm). It was hypothesized that if a correlation could be identified between the functional status of the muscles studied and the magnitude of pelvic rotation, this could contribute to more effective targeting of therapeutic measures aimed at restoring symmetrical pelvic position. This could have important implications in relation to the role of spatial shaping of the pelvis in the occurrence of dysfunctions and diseases associated with its asymmetrical position [11,12,15,42,43,44]. However, the results obtained did not clearly confirm the assumption that there is such a relationship. This is because the presence of differences in bilateral muscle stiffness in subjects with symmetrical and asymmetrical pelvis was not confirmed. The only differentiation that was expected to be observed was for the gluteus maximus muscles, where slightly lower muscle stiffness was found on the right side. However, this differentiation was above the assumed threshold of statistical significance (*p* = 0.0698). Additionally, no significant correlations were identified between the amount of pelvic rotation and the stiffness of the muscles studied by tensiomyography. In addition, previous studies by the authors showed no direct relationship between the stiffness of the muscles stabilizing the LPH complex and the amount of pelvic tilt in the sagittal plane [45]. The lack of a relationship between the magnitude of pelvic asymmetry and bilateral functional variation in the muscles of the lateral abdominal wall using ultrasound imaging was also confirmed by Biały et al. [46]. When we analyze the results of our own study and those obtained by Bialy, it is not possible to clearly determine whether the pelvis rotates due to changes in the functional state of the muscles that stabilize the pelvis, or whether the pelvis rotates due to other causes and the functional state of the muscles is a consequence of changes in the spatial positioning of the iliac crest. Determining the causes of pelvic rotation is beyond the scope of this paper. However, for a better understanding of the issue, it may be helpful to cite here some concepts that attempt to explain the causes of pelvic rotation. This is especially relevant in the context of selecting therapeutic methods used in cases of observed asymmetries. Lewit describes the mechanism of pelvic torsion as an alternating asymmetry of the two pelvic bones, in which an elevation of the posterior superior iliac spine is accompanied by a lowering of the anterior superior iliac spine on the same side. It is often observed that there is a lowering of the superior frontal knees on the right side. The mechanism described by Lewit is thought to cause oblique positioning of the sacrum, which may lead to asymmetry of the base for the spine and asymmetric transfer of dynamic loads from the lower extremities to the trunk. It is thought that these changes are reflexive in nature and their primary cause is a disturbance at the level of the junction of the cranial and cervical spine [19]. Ackermann suggests that the asymmetry may be the result of an external force or sudden overloading of one of the lower extremities, and that there may be a twisting of the pelvic bone in a clockwise or counterclockwise direction. This, in turn, may lead to an increase in muscle tone, which can sometimes be mistaken for limb shortening [47]. The loading mechanism for the formation of functional asymmetry of the LPH complex is also pointed out by Gnat [48,49]. According to Savory and Kaute [50], there appears to be a physiological basis for the appearance of functional pelvic torsion. It is evident that due to the lateralization of the body, there is a peculiar functional specialization of the lower limbs. This specialization can be defined as follows: one leg (the dominant one) plays a supporting role, while the other leg plays a motor role. It has been observed that right-legged individuals tend to place more weight on their right leg, which they utilize in situations requiring greater force release. In contrast, the left leg is more adept at leaping. It is also used for bouncing. It has been suggested that prolonged loading of one limb may potentially lead to an asymmetrical alignment of the two hip bones relative to each other, where one side of the hip bone may rotate upward and backward, while the other side may rotate downward and forward. This can potentially result in a perceived shortening of the lower limb on the side of the posterior rotation. Other possible causes of hip rim asymmetry may be rotated body patterns, which can result in fascial tension in specific body parts, potentially leading to asymmetry in the lumbopelvic complex [51]. Torsional Upper Crossed Syndrome (TUCS) has been described by Morris et al. [52]. Kouwenhoven et al. [53] observed what appeared to be a tendency for the high thoracic vertebrae to rotate to the left, and for the middle and lower thoracic vertebrae to rotate to the right. This observation may possibly offer a potential explanation for the subsequent rotation of the pelvis to the right side, which could be a contributing factor to the observed asymmetry in pelvic floor muscles. It has been hypothesized that the asymmetrical range of motion of the hip joints and pelvis may result in alterations to muscle and tendon length and function [54]. In addition to the mechanism of pelvic rotation, the authors of the study encountered a second difficulty, namely the assessment of muscle stiffness. The definition of stiffness, and the methods of measuring it, are not entirely clear and could be clarified. The variability of opinions and the lack of a clear definition of muscle tension mean that clarification is always needed when examining muscle tension as to how the term should be understood [55,56]. In the majority of cases, the focus is on passive or dynamic stiffness [57]. The assessment of passive stiffness typically involves the implementation of methodologies that are founded upon elastographic, myotonometric and tensiomyographic measurements, in conjunction with clinical evaluations. However, it should be noted that these methods study and analyze different aspects of tissue stiffness, which makes it difficult to compare results [31,39,55,58,59]. In the context of TMG analysis, the calculation of stiffness is indirect, with its interpretation being based on the measurement of maximum radial displacement (Dm) [60]. Myotonometry, on the other hand, defines stiffness differently, as the relationship between the force produced by a mechanical impulse and the depth of tissue deformation [61]. If we may make a suggestion, it might be beneficial to consider expanding therapy for muscle imbalance to include the assessment of the function of higher-order mechanisms controlling muscle tension at the level of the central nervous system, in particular the pontomedullary reticular formation (PMRF) [62,63,64].

This study did not confirm the relationship between changes in the stiffness of the muscles stabilizing the LPH complex and changes in their geometric position. Therefore, we cannot unequivocally conclude that, in light of the results obtained, the existing methods used to restore muscle–fascia balance and achieve a symmetrical pelvic position are ineffective or inappropriate. We can only conclude that tensiomyographic studies of the stiffness of the muscles isolated in the muscle study did not show direct correlations with the amount of rotation of the hip girdle.

### Study Limitations

The authors are aware that the most significant limitation in determining the relationship between the functional state of the muscles and, in particular, their stiffness, and the asymmetry of the pelvic position is the absence of a precise definition of the concepts of muscle tone and muscle stiffness. The assessment of the relationship between changes in pelvic position and muscle stiffness, as defined by tensiomyography, is limited to the presence or absence of a maximal displacement (Dm) or their contraction time parameters, Td and Tc. To ascertain a definitive relationship between stiffness and pelvic rotation, the study should be expanded to encompass additional methods for evaluating stiffness in other domains, as well as muscle strength and length. A further limitation of the study methodology was the inability to reach deeper muscles, for instance, through elastography. The exclusive inclusion of women in the study group may also have been a constraint. While it is true that one of the conditions for inclusion was the absence of periods and pregnancy, it should be noted that changes in muscle stiffness can also occur during other periods of the menstrual cycle. The study’s homogeneity, in terms of the gender of the respondents, may also be considered a strength, despite appearing to be a limitation. Another strength of the study is the use of tensiomyography, a method that is gaining acceptance as a research tool. This study also employs an anthropological inclinometer of a bespoke design, in conjunction with a clinical classification of pelvic asymmetry that is original to the study.

## 5. Conclusions

The tensiomyography examinations did not show any significant differences in the stiffness of muscles in respondents with a symmetric or rotated pelvis. There were also no bilateral differences in muscle functions in the participants with the diagnosed pelvic rotation. Additionally, no clear linear correlations were observed between the amount of pelvic rotation and the tensiomyography variables studied.

## Figures and Tables

**Table 1:** Research group.

Variable	SP *n* = 20	RP *n* = 20
	Mean ± Std.	Mean ± Std.
Weight (kg)	57.0 ± 4.01	59.7 ± 5.01
Height (cm)	164.4 ± 5.35	167.9 ± 5.74
BMI (kg m^−2^)	21.2 ± 1.59	21.2 ± 1.71

SP—symmetric pelvis; RP—asymmetric (rotated) pelvis; *n*—number of participants; Std—standard deviation.

**Table 2:** Descriptive analysis and analysis of differences in contraction time, Tc (ms), and displacement, Dm (mm), of the LPHC stabilizing muscles (whole group).

Muscles	DM (mm)		
	Left Side *n* = 40	Right Side *n* = 40	*p*
	Mean ± Std.	Mean ± Std.	
Gluteus maximus	5.0 ± 2.57	6.4 ± 3.12	0.061
Biceps femoris	4.4 ± 2.38	4.3 ± 2.12	0.856
Rectus femoris	5.6 ± 2.46	5.5 ± 2.70	0.848
Rectus abdominis	4.2 ± 2.06	4.4 ± 2.10	0.724
Obliques abdominis ext.	2.8 ± 1.70	2.6 ± 1.83	0.657
Erector spinae	3.3 ± 1.42	3.4 ± 1.47	0.810

*n*—number of participants; Std.—standard deviation; *p*—*p*-value (*t*-test).

**Table 3:** Descriptive analysis and analysis of differences in displacement, Dm (mm), of the LPHC stabilizing muscles in women with a symmetric or rotated pelvis (symmetric pelvis group *n* = 20, rotated pelvis group *n* = 20).

Muscles	Variable	Type of Pelvis	Left Side Mean ± Std.	Right Side Mean ± Std.	*p*
Gluteus maximus	Dm	SP	5.5 ± 3.67	6.5 ± 4.07	0.532
		RP	4.9 ± 2.01	6.3 ± 2.65	0.0698
	*p*		0.555	0.869	
Biceps femoris	Dm	SP	4.4 ± 2.41	4.6 ± 2.37	0.845
		RP	4.3 ± 2.16	4.2 ± 1.85	0.874
	*p*		0.8855	0.584	
Rectus femoris	Dm	SP	5.6 ± 3.16	5.6 ± 3.44	0.965
		RP	5.9 ± 2.25	5.6 ± 2.56	0.723
	*p*		0.713	0.986	
Rectus abdominis	Dm	SP	4.7 ± 2.34	4.8 ± 2.58	0.886
		RP	4.2 ± 2.10	4.4 ± 1.90	0.799
	*p*		0.516	0.532	
Obliques abdominis ext.	Dm	SP	2.8 ± 1.93	2.9 ± 1.66	0.922
		RP	2.8 ± 1.71	2.6 ± 2.10	0.851
	*p*		0.939	0.745	
Erector spinae	Dm	SP	3.3 ± 0.99	3.3 ± 1.38	0.920
		RP	3.3 ± 1.65	3.7 ± 1.43	0.449
	*p*		0.981	0.438	

*p*—*p*-value (*t*-test); SP—symmetric pelvis; RP—rotated pelvis.

**Table 4:** Pearson’s correlation between the pelvic rotation magnitude and displacement (Dm).

Muscle	Variable	
	Dm [mm]	
	Left Side	Right Side
Gluteus maximus	r = −0.076 *p* = 0.763	r = −0.123 *p* = 0.627
Biceps femoris	r = 0.198 *p* = 0.416	r = 0.065 *p* = 0.793
Rectus femoris	r = −0.358 *p* = 0.132	r = −0.334 *p* = 0.162
Rectus abdominis	r = 0.060 *p* = 0.807	r = 0.108 *p* = 0.659
Obliques abdominis ext.	r = 0.029 *p* = 0.905	r = 0.002 *p* = 0.993
Erector spinae	r = −0.416 *p* = 0.076	r = −0.354 *p* = 0.137

Dm—displacement; r—Pearson’s correlation; *p*—*p*-value.

## Data Availability

The minimal data set is contained within our paper.
